# An African perspective on the genetic risk of chronic kidney disease: a systematic review

**DOI:** 10.1186/s12881-018-0702-x

**Published:** 2018-10-19

**Authors:** Cindy George, Yandiswa Y Yako, Ikechi G Okpechi, Tandi E Matsha, Francois J. Kaze Folefack, Andre P Kengne

**Affiliations:** 1 Non-Communicable Diseases Research Unit, South African Medical Research Council, Parow Valley, PO Box 19070, Cape Town, South Africa; 2 Department of Human Biology, Faculty of Health Sciences, Walter Sisulu University, Mthatha, South Africa; 3 Department of Medicine, Division of Nephrology and Hypertension, University of Cape Town, Cape Town, South Africa; 4 Kidney and Hypertension Research Unit, University of Cape Town, Cape Town, South Africa; 5 Department of Biomedical Sciences, Faculty of Health and Wellness Science, Cape Peninsula University of Technology, Bellville, Cape Town, South Africa; 6 Faculty of Medicine and Biomedical Sciences, University of Yaounde I, Yaounde, Cameroon; 7 Medicine Unit, Yaounde University Teaching Hospital, Yaounde, Cameroon

**Keywords:** Chronic kidney disease, End-stage renal disease, Genetics, Africa

## Abstract

**Background:**

Individuals of African ethnicity are disproportionately burdened with chronic kidney disease (CKD). However, despite the genetic link, genetic association studies of CKD in African populations are lacking.

**Methods:**

We conducted a systematic review to critically evaluate the existing studies on CKD genetic risk inferred by polymorphism(s) amongst African populations in Africa. The study followed the HuGE handbook and PRISMA protocol. We included studies reporting on the association of polymorphism(s) with prevalent CKD, end-stage renaldisease (ESRD) or CKD-associated traits. Given the very few studies investigating the effects of the same single nucleotide polymorphisms (SNPs) on CKD risk, a narrative synthesis of the evidence was conducted.

**Results:**

A total of 30 polymorphisms in 11 genes were investigated for their association with CKD, ESRD or related traits, all using the candidate-gene approach. Of all the included genes, *MYH9, AT1R* and *MTHFR* genes failed to predict CKD or related traits, while variants in the *APOL1, apoE, eNOS, XPD, XRCC1, renalase, ADIPOQ,* and *CCR2* genes were associated with CKD or other related traits. Two SNPs (rs73885319, rs60910145) and haplotypes (G-A-G; G1; G2) of the apolipoprotein L1 (*APOL1*) gene were studied in more than one population group, with similar association with prevalent CKD observed. The remaining polymorphisms were investigated in single studies.

**Conclusion:**

According to this systematic review, there is currently insufficient evidence of the specific polymorphisms that poses African populations at an increased risk of CKD. Large-scale genetic studies are warranted to better understand susceptibility polymorphisms, specific to African populations.

**Electronic supplementary material:**

The online version of this article (10.1186/s12881-018-0702-x) contains supplementary material, which is available to authorized users.

## Background

Chronic kidney disease (CKD) is fast becoming a leading public health issue in Africa, with an estimated prevalence of 14.3% in the general population, and 36.1% in high-risk populations [1]. Due in part to increasing rates of type 2 diabetes, hypertension and obesity, the prevalence of CKD continues to rise [2]. However, marked variability in the incidence of CKD between population groups, suggests additional factors contributing to CKD aetiology [3]. Indeed, prevalent end-stage renal disease (ESRD), which is the terminal stage of CKD, is 4-fold higher among African ethnicity as compared to European ethnicity [4, 5] and individuals of African ethnicity progress faster from moderately decreased kidney function to ESRD [6]; thus highlighting African ethnicity as a contributing risk factor for CKD [4, 5].

Over the past decade, through the use of genome-wide association studies (GWAS), researchers have identified various genomic regions with common genetic variants associated with CKD traits [7]. However, a limitation of the majority of GWAS’s conducted to date is the paucity of studies conducted in individuals of African ancestry and even less in Africans living in Africa [7–10]. Despite, Africa being one of the most ethnically and genetically diverse regions of the world [11], these populations are understudied, with most of the common loci associated with CKD in non-African populations not being replicated in African populations. Though African migrants living in Europe and America are genetically linked with African ancestry [12, 13], these genetic variants cannot be extrapolated to Africans residing in Africa. This is mainly due to genetic admixture of American and European populations, as well as differences in environment, cultural and lifestyles [11]. Accordingly, identification of genetic loci for CKD in African populations will help to advance our understanding of the underpinnings of CKD in individuals of African descent.

There is currently no systematic review evaluating the CKD-associated genes found in African populations residing in Africa. The main purpose of this review is thus to critically evaluate the existing studies on CKD genetic risk inferred by polymorphisms amongst African populations in Africa, and explore the specific effect these genetic loci have on CKD development in the African population.

## Methods

### Protocol and registration

The review was conducted using the Preferred Reporting Items for Systematic Reviews and Meta-Analysis PRISMA framework [14] and HuGENET™ HuGE Review handbook [15]. The methods of the analysis and inclusion criteria were specified in advance and documented in a protocol in the PROSPERO database (registration number: CRD42017058440).

### Selection of eligible studies, types of studies and sources of information

Relevant studies published until August 2017 were identified through a comprehensive electronic search of major databases such as MEDLINE (via PubMed)*,* EBSCOhost, Scopus, and Web of Science, using an African search filter [16] and without any starting date or language restrictions. Medical Subject Headings (MeSH) terms and Boolean operators, such as AND/OR/NOT, were used to string terms together (refer to Additional files 1, 2, 3 and 4: Tables S1–S4). Publication bibliographies were searched to further enhance the search strategy.

### Data collection

**Fig. 1:**
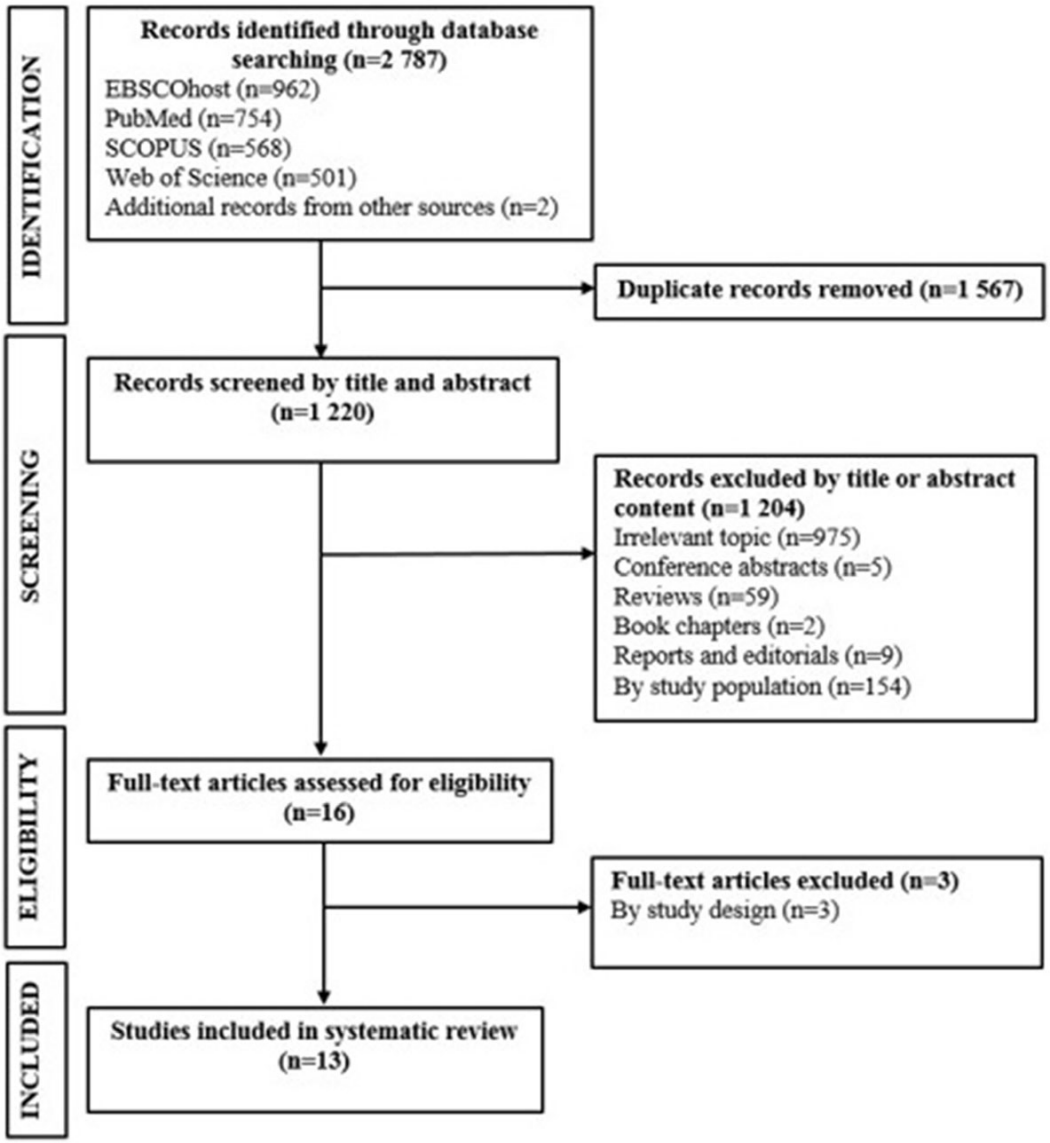
Selection process for studies included in the systematic review

Two authors (CG and YYY) independently conducted the database searches and sequentially (titles, abstracts and then full texts) screened them for inclusion (Fig. 1). In situations of disagreements between the two authors, a third author (APK) arbitrated for eligibility. The inclusion criteria was that a study had to be an original study containing independent data that were obtained from case-control or cohort studies, which specifically conducted genetic association analyses on African populations residing in Africa. These studies had to report on study population characteristics, methods, CKD or renal traits (such as serum creatinine, estimated glomerular filtration rate (eGFR), urinary albumin excretion), genes and polymorphisms, genotyping technique(s), statistical analyses, and report on allele and genotype frequencies. Studies were excluded if, [1] the conducted analyses were exclusively on migrant African populations, [2] the entire cohort consisted of only high-risk individuals (a population of only type 2 diabetic or hypertensive patients), [3] the study did not report the estimate effects and/or *p*-values, allele and genotype frequencies, and if [4] the study was a meta-analysis, review or any other form of publication that do not have primary data. Full articles were obtained for all abstracts and titles that met inclusion criteria as well as those that certainty of inclusion was unclear. The two authors (CG and YYY) screened the full-text articles, and selected full manuscripts according to the inclusion criteria. Disagreements were resolved through discussion or if consensus were not met, reviewed by a third author (APK). The reasons for excluding studies were also recorded.

### Data extraction, assessment and synthesis

The data extracted from selected articles included the name of the first author and year of publication, study setting and design, population characteristics, genetic models used for measures of association, adjustment (if any) for confounding variables, allele and genotype frequencies, and the study outcome. Data extraction was done by one author (CG), and another author (YYY) verified the accuracy and validity of extracted data. As recommended by Sagoo et al. [17], we assessed the existence of bias considering the following: case definition, population stratification, reporting of methods used (sample size of a study population, genotyping method and its reliability/accuracy, validation of results, statistical analyses). Given the very few studies investigating the effects of the same SNPs on CKD risk across different settings/countries, attempting to pool studies were deemed meaningless, thus, we opted to conduct a narrative synthesis of the evidence instead of a meta-analysis.

## Results

### Study selection

We retrieved 2787 citations (962 from EBSCOhost; 754 from MEDLINE; 568 from SCOPUS; 501 from Web of Science; 2 from publication bibliography) from our searches. Of these, 2771 citations were not eligible for inclusion for the following reasons: duplicate (*n* = 1567) or irrelevant to this review based on the title or abstract (*n* = 1204). Consequently, 16 full-text articles were reviewed and of those, three citations were excluded, based on not meeting the inclusion criteria of this review, resulting in 13 eligible articles retained for the systematic review (Fig. 1).

### Characteristics of included studies

Table 1 describes the characteristics of the genetic studies included in this review. All the studies were conducted between 2009 and 2016 with the vast majority conducted in Egypt (*n* = 7), followed by Nigeria and South Africa (*n* = 2, each), and Morocco and Tunisia each with only one reported study on CKD genetic association. Overall, nine studies (69.2%) were from three north-African countries [18–26] and the rest from sub-Saharan African countries [27–30]. The study population ranged from 87 to 859 participants per study, with the mean age ranging from 8.7 to 58.9 years and a male predominance in all except the two South African studies, where only 22–23% were male [27, 28]. Of the thirteen studies included, kidney dysfunction was characterized mainly by an estimated glomerular filtration rate (eGFR) equal to or less than 60 ml/min/1.73m^2^ [18, 23, 25, 27, 28]. The remaining studies used other surrogate measures to determine kidney dysfunction, which included ESRD (undergoing haemodialysis) [19, 21, 22, 24, 26], elevated serum creatinine levels [20] and a combination of serum creatinine levels greater or equal to 170 μmol/l and dipstick proteinuria greater or equal to 2 [30] or serum creatinine above 1.4 mg/dl (men) and 1.2 mg/dl (women) and urinary albumin to creatinine ratio (ACR) above 30 mg/g [29]. The CKD patients included in these studies were of different aetiologies, reflective of the diversity in nephropathy present in Africa.

**Table 1:** Characteristics of genetic studies conducted in Africa

Authors	Study design	Country	Population	Sample size (case/control)	Mean age (years±SD)	Male (%) (case/control)	Measure of kidney dysfunction	Type of nephropathy
Tayo et al. [29]	Case-control	Nigeria	Yoruba tribe	87/79	42.1 ± 16.9 (case) 35.2 ± 8.2 (control)	53/51	*Serum creatinine* (> 1.4 mg/dl, men; > 1.2 mg/dl, women) *Spot urine* (ACR > 30 mg/g)	Hypertension-associated (50.5%) HIV-associated (9.2%) Proteinuric (40.2%)
Ulasi et al. [30]	Case-control	Nigeria	Igbo tribe	44/43	46.6 ± 17.8 (case) 42.7 ± 10.9 (control)	57/63	Serum creatinine (≥170 μmol/l or proteinuria ≥2+)	HIV-associated (18.2%) NS (81.8%)
Matsha et al. [28]	Cross-sectional	South Africa	Mixed-race	*By MDRD* 68/648 *By CKD-EPI* 67/649	53.6 ± 14.9 (total)	22.1 (total)	eGFR (< 60 ml/min/1.73m^2^ based on MDRD and CKD-EPI equations)	NS
Matsha et al. [27]	Cross-sectional	South Africa	Mixed-race	*By MDRD* 79/780 *By CKD-EPI* 73/786	53.1 ± 14.1 (total)	22.7 (total)	eGFR (< 60 ml/min/1.73m^2^ based on MDRD and CKD-EPI equations)	NS
Lahrach et al. [21]	Case-control	Morocco	NS	109/97	44.9 ± 14.4 (case) 46.8 ± 11.8 (control)	NS	ESRD undergoing haemodialysis	NS
Hanna et al. [19]	Case-control	Egypt	NS	50/44	37.9 ± 14.3 (case) NS (control)	64/NS	ESRD undergoing haemodialysis	Diabetic nephropathy (26%) Hypertensive nephrosclerosis (22%) Systemic lupus erythematosus (8%) Polycystic kidney disease (10%) Idiopathic (34%)
Kerkeni et al. [20]	Case-control	Tunisia	NS	100/120	51.0 ± 15.0 (case) 54.0 ± 10.0 (control)	55/73	Serum creatinine (thresholds NS; groups included MRF, SRF and ESRD)	Non-diabetes CKD with the following aetiologies: Chronic glomerular nephritis (41%) Chronic tubulointerstitial nephropathy (30%) Vascular nephropathy (23%) Idiopathic (6%)
Elshamaa et al. [18]	Case-control	Egypt	NS	78/30	9.14 ± 7.59 (CT); 10.62 ± 3.49 (MHD) (case) 8.7 ± 4.51 (control)	51/67	eGFR (according to K/DOQ1 guidelines): *Undergoing CT* GFR (range, 15–29 ml/min/1.73m^2^) *Undergoing MHD* GFR (range, 5–15 ml/min/1.73m^2^)	Advanced CKD with the following aetiology: Renal hypoplasia/dysplasia (20.5%) Obstructive uropathies (17.9%) Neurogenic bladder (7.7%) Metabolic (2.6%) Hereditary nephropathies (21.8%) Glomerulopathy (2.6%) Idiopathic (26.9%)
Radwan et al. [22]	Case-control	Egypt	NS	98/102	47.8 ± 14.2 (total)	50/56	ESRD undergoing hemodialysis	Hypertension-associated (44.9%) Diabetes-associated (11.2%) Preeclampsia (4%) Drug-induced (3%) Glomerulonephritis (6.1%) Obstructive uropathy (5.1%) Atrophic kidney (3%) Systemic lupus erythematosus (5.1%) Polycystic kidney (2%) Combined polycystic kidney and hypertension (1%) Combined DM and hypertension (6.1%) Amyloidosis and hypertension (1%) Idiopathic (7.1%)
Rezk et al. [23]	Case-control	Egypt	NS	178 (83 NT; 95 HT)/ 178	47.4 ± 9.3 (case) NS (control)	NS	eGFR (according to K/DOQ1 guidelines)	Hypertension-associated (53.4%) NS (46.6%)
Abdallah et al. [26]	Case-control	Egypt	NS	139/50	NS	48.2/NS	ESRD undergoing hemodialysis	NS
Elshamaa et al. [25]	Case-control	Egypt	NS	78/70	9.14 ± 7.59 (CT); 10.62 ± 3.49 (MHT) (case) 10.7 ± 4.51 (control)	51/57	eGFR (according to K/DOQ1 guidelines): *Undergoing CT* GFR (range, 15–29 ml/min/1.73m^2^) *Undergoing MHD* GFR (range, 5–15 ml/min/1.73m^2^)	Advanced CKD with the following aetiology: Renal hypoplasia/dysplasia (20.5%) Obstructive uropathies (17.9%) Neurogenic bladder (7.7%) Metabolic (2.6%) Hereditary nephropathies (21.8%) Glomerulopathy (2.6%) Idiopathic (26.9%)
Elhelbawy et al. [24]	Case-control	Egypt	NS	70/30	60.2 ± 9.4 (case) 58.9 ± 10.7 (control)	61.4/63.3	ESRD undergoing hemodialysis	NS

*ACR* albumin/creatinine ratio, *CKD* chronic kidney disease, *CKD-EPI* Chronic Kidney Disease Epidemiology Collaboration, *CT* conservative treatment, *DM* diabetes mellitus, *eGFR* estimated glomerular filtration rate, *ESRD* end-stage renal disease, *HT* hypertensive, *K/DOQI* NKF Kidney Disease Outcomes Quality Initiative, *MDRD* Modification of Diet in Renal Disease, *MHD* maintenance hemodialysis, *MRF* moderate renal failure, *NS* not specified, *NT* normotensive, *PCR* polymerase chain reaction, *RFLP* restriction fragment length polymorphism, *SRF* severe renal failure, *SSA* sub-Saharan Africa

Table 2 summarizes the polymorphisms investigated in the included studies. Thirty different polymorphisms (including SNP, indels and repeats) in 11 genes have been studied in various population groups in Africa. Of the polymorphisms investigated by selected studies, only three SNPs of the *APOL1* gene (rs73885319, rs60910145, rs71785313) were studied in more than one population group, which included the Yoruba [29] and Igbo [30] tribes of Nigeria and the South African mixed-race population group [27]. The remaining 27 polymorphisms of the *MYH9*, *apoE, AT1R, eNOS, MTHFR, XPD, XRCC1, renalase, ADIPOQ* and *CCR2* genes were each studied in only one ethnic group. Eight of the included genetic association studies assessed the distribution of allele frequency by formally testing for Hardy-Weinberg equilibrium (HWE), and one study assumed HWE without formal testing [21]. Of those formally tested, only one polymorphism showed a departure from HWE (*MYH9* rs4821480), and was subsequently removed from further association analysis in that study [28]. Adjustment for confounders was not consistent across studies, with six studies not providing information on the degree of adjustment or variables accounted for [21–26]. The remaining seven studies all adjusted for at least age and gender [18–20, 27–30]. In all studies, the genomic DNA was extracted from whole blood samples and genotyped by methods including TaqMan genotyping assays, polymerase chain reaction restriction fragment length polymorphism (PCR-RFLP) and gel-electrophoresis and confirmed by PCR-sequencing.

**Table 2:** Polymorphisms investigated in African studies

Author	Gene (chromosome region)	Polymorphism	Minor allele frequency: case/control (%)	Genotyping method	HWE	Adjustment	Effect estimate OR/ HR (95% CI)	Outcome
Tayo et al. [29]	*APOL1* (22q12.3)	rs9622363	A: 25.86/29.75	Custom Fluidigm ™ 96.96 array platform; TaqMan genotyping assay	0.788	Age Gender	OR (additive): 0.76 (0.45 to1.31); *p* = 0.875 OR (dominant): 0.88 (0.47 to 1.66); *p* = 0.999 OR (recessive): 0.24 (0.05 to 1.29); *p* = 0.377	CKD
		rs73885319	A: 44.19/26.58		1.00		OR (additive): 2.29 (1.39 to 3.77); *p* = 0.005 OR (dominant): 2.59 (1.34 to 5.00); *p* = 0.025 OR (recessive): 3.85 (1.31 to 11.36); *p* = 0.038	
		rs60910145	G: 50.00/30.13		0.114		OR (additive): 2.04 (1.32 to 3.17); *p* = 0.006 OR (dominant): 2.54 (1.31 to 4.92); *p* = 0.034 OR (recessive): 3.12 (1.35 to 7.20); *p* = 0.015	
		G2: rs71785313	D: 8.62/12.66		1.00		OR (additive): 0.61 (0.29 to 1.31); *p* = 0.701 OR (dominant): 0.64 (0.29 to 1.40); *p* = 0.816 OR (recessive): NS	
		G1: rs73885319 and rs60910145	44.19/26.92 (A-G haplotype)				OR (additive): 2.25 (1.36 to 3.71); *p* = 0.005 OR (dominant): 2.52 (1.30 to 4.88); *p* = 0.051 OR (recessive): 3.80 (1.29 to 11.22); *p* = 0.026	
			50.00/69.87 (G-T haplotype)				OR (additive): 0.49 (0.32 to 0.76); p = 0.005 OR (dominant): 0.32 (0.14 to 0.73); *p* = 0.018 OR (recessive): 0.40 (0.21 to 0.77); *p* = 0.031	
	*MYH9* (22q12.3)	rs11912763	A: 38.51/27.22		1.00		OR (additive): 1.68 (1.02 to 2.76); *p* = 0.197 OR (dominant): 2.03 (1.06 to 3.87); *p* = 0.183 OR (recessive): 1.70 (0.58 to 4.94); *p* = 0.872	
		rs2032487	T: 18.39/26.28		0.770		OR (additive): 0.68 (0.40 to 1.16); *p* = 0.580 OR (dominant): 0.64 (0.33 to 1.23); *p* = 0.645 OR (recessive): 0.55 (0.14 to 2.22); *p* = 0.934	
		rs4821481	T: 18.39/26.58		0.777		OR (additive): 0.66 (0.39 to 1.13); *p* = 0.532 OR (dominant): 0.61 (0.32 to 1.18); *p* = 0.583 OR (recessive): 0.55 (0.14 to 2.24); *p* = 0.940	
		rs5750248	C: 25.86/36.08		1.00		OR (additive): 0.61 (0.37 to 0.99); *p* = 0.225 OR (dominant): 0.56 (0.29 to 1.05); *p* = 0.354 OR (recessive): 0.46 (0.15 to 1.41); *p* = 0.627	
		rs5750250	A: 26.16/37.97		0.635		OR (additive): 0.56 (0.34 to 0.94); *p* = 0.141 OR (dominant): 0.51 (0.27 to 0.97); *p* = 0.208 OR (recessive): 0.44 (0.14 to 1.38); *p* = 0.576	
Ulasi et al. [30]	*APOL1* (22q12.3)	G1: rs73885319 and rs60910145 G2: rs71785313	59/30 20/23	PCR-sequencing; PCR-RFLP	NS	Age Gender BMI HIV	OR: 4.8 (1.6 to 14.9); p = 5.1E-03	CKD
Matsha et al. [28]	*MYH9* (22q12.3)	rs5756152	G: 12.4 (overall)	PCR-sequencing; TaqMan genotyping assay	> 0.999	Age Gender Diabetes ACR	OR (additive): − 2.3 (− 5.6 to 0.9); *p* = 0.16 OR (additive): 1.91 (− 1.32 to 5.15); *p* = 0.25 OR (additive): 1.83 (− 1.23 to 4.89); *p* = 0.24 OR (additive): − 1.6 (− 18.9 to 15.6); *p* = 0.85	Serum creatinine eGFR(MDRD) eGFR (CKD-EPI) ACR
		rs4821480	T: 30.3 (overall)		0.053		NS	
		rs12107	A: 22.2 (overall)		0.908		OR (additive): 0.4 (− 2.2 to 2.9); *p* = 0.78 OR (additive): − 0.07 (− 2.61 to 2.46); *p* = 0.95 OR (additive): 0.13 (− 2.27 to 2.54); *p* = 0.91 OR (additive): 1.0 (− 12.6 to 14.5); *p* = 0.90	Serum creatinine eGFR(MDRD) eGFR (CKD-EPI) ACR
Matsha et al. [27]	*APOL1* (22q12.3)	rs73885319	G: 3.6 (overall)	PCR-sequencing; TaqMan genotyping assay	0.150	Age Gender Diabetes Hypertension	OR (additive): -0.018 (-0.069 to 0.0034); p=0.503 OR (dominant): -0.026 (-0.080 to 0.028); p=0.341 OR (recessive): 0.191 (-0.094 to 0.478); p=0.189	Serum creatinine
							OR (additive): 0.99 (-4.42 to 6.40); p=0.720 OR (dominant): 1.75 (-9.93 to 7.44); p=0.546 OR (recessive): -18.54 (-48.59 to 11.51); p=0.227	eGFR(MDRD)
							OR (additive): 2.07 (-2.40 to 6.55); p=0.364 OR (dominant): 2.96 (-1.74 to 7.66); p=0.217 OR (recessive): -18.90 (-43.76 to 5.96); p=0.136	eGFR (CKD-EPI)
							OR (additive): 0.76 (0.27 to 2.16); p=0.601 OR (dominant): 0.56 (0.18 to 1.79); p=0.307 OR (recessive): 23.47 (0.92 to 599.29); p=0.074	CKD (MDRD)
							OR (additive): 1.08 (0.38 to 3.03); p=0.887 OR (dominant): 0.81 (0.26 to 2.54); p=0.720 OR (recessive): 42.72 (1.22 to ∞); p=0.047	CKD (CKD-EPI)
							OR (additive): -0.126 (-0.446 to 0.195); p=0.442 OR (dominant): -0.096 (-0.436 to 0.245); p=0.583 OR (recessive): -1.02 (-2.62 to 0.57); p=0.210	ACR
		rs60919145	G: 3.4 (overall)		0.127		OR (additive): -0.020 (-0.072 to 0.033); p=0.466 OR (dominant): -0.029 (-0.084 to 0.026); p=0.307 OR (recessive): 0.192 (-0.094 to 0.478); p=0.289	Serum creatinine
							OR (additive): 1.26 (-4.27 to 6.79); p=0.656 OR (dominant): 2.09 (-3.73 to 7.91); p=0.482 OR (recessive): -18.54 (-48.59 to 11.51); p=0.227	eGFR(MDRD)
							OR (additive): 2.28 (-2.29 to 6.86); p=0.328 OR (dominant): 3.24 (-1.57 to 8.06); p=0.187 OR (recessive): -18.90 (-43.76 to 5.96); p=0.136	eGFR (CKD-EPI)
							OR (additive): 0.80 (0.28 to 2.27); p=0.665 OR (dominant): 0.59 (0.18 to 1.89); p=0.350 OR (recessive): 23.47 (0.92 to 599.29); p=0.074	CKD (MDRD)
							OR (additive): 1.12 (0.39 to 3.16); p=0.836 OR (dominant): 0.84 (0.27 to 2.65); p=0.767 OR (recessive): 42.72 (1.22 to ∞); p=0.047	CKD (CKD-EPI)
							OR (additive): -0.178 (-0.504 to 0.147); p=0.283 OR (dominant): -0.154 (-0.502 to 0.193); p=0.384 OR (recessive): -1.02 (-2.62 to 0.57); p=0.210	ACR
		rs71785313	Del: 5.8 (overall)		0.420		OR (additive): 0.019 (-0.022 to 0.060); p=0.367 OR (dominant): -0.020 (-0.024 to 0.064); p=0.382 OR (recessive): 0.038 (-0.144 to 0.219); p=0.684	Serum creatinine
							OR (additive): -2.38 (-6.68 to 1.93); p=0.323 OR (dominant): -2.35 (-6.99 to 2.30); p=0.323 OR (recessive): -7.16 (-26.19 to 11.87); p=0.461	eGFR(MDRD)
							OR (additive): -2.91 (-6.46 to 0.65); p=0.110 OR (dominant): -3.03 (-6.88 to 0.81); p=0.123 OR (recessive): -5.99 (-21.74 to 9.76); p=0.456	eGFR (CKD-EPI)
							OR (additive): 0.86 (0.39 to 1.93); p=0.712 OR (dominant): 0.91 (0.38 to 2.14); p=0.823 OR (recessive): 0.0	CKD (MDRD)
							OR (additive): 1.00 (0.42 to 2.34); p=0.993 OR (dominant): 1.07 (0.43 to 2.66); p=0.890 OR (recessive): 0.0	CKD (CKD-EPI)
							OR (additive): 0.035 (-0.207 to 0.277); p=0.777 OR (dominant): 0.050 (-0.214 to 0.314); p=0.710 OR (recessive): -1.123 (-1.134 to 0.888); p=0.811	ACR
Lahrach et al. [21]	*apoE* (19q13.32)	e2 (rs7412-T, rs429358-T)	3.0/6.0	PCR-sequencing; gelelectrophoresis	NS	None	OR (NS): 0.473 (0.181 to 1.235); p=0.093	ESRD
		e3 (rs7412-C, rs429358-T	73.0/82.0 (Reference)				Reference group	
		e4 (rs7412-C, rs429358-C)	24.0/12.0				OR (NS): 0.491 (0.277 to 0.870); p=0.009 (UA)	
Hanna et al. [19]	*AT1R* (3q24)	A1166C	C: 86.0/83.0	PCR-RFLP	NS	Age Gender	HR (NS): 1.254 (0.658 to 2.389); p=0.491	ESRD
Kerkeni et al. [20]	*eNOS* (7q36.1)	G894T (exon7)	T: 27.0/22.1	PCR-RFLP	Satisfied HWE (p-value NS)	Age Gender Smoking Hypertension Dyslipidaemia Cholesterol Homocysteine MTHFR C677T eNOS G894T	EE not reported; p=0.028 (difference in allele frequency)	CKD
Elshamaa et al. [18]	*eNOS* (7q36.1)	4a (intron4)	CT and MHD/controls: 32.8 and 33.7/22.7 CT and MHD/controls: 67.2 and 66.3/78.3	PCR-sequencing; gelelectrophoresis	Satisfied HWE (p-value NE)	Age Hypertension SBP DBP Serum NO	EE not reported; p<0.05 (patient groups vs control)	Advanced CKD (ESRD)
Radwan et al. [22]	*XPD* (9)	Asp312Asn	Asn: 35.0/36.0	PCR-RFLP	Satisfied HWE (p-value NE)	NS	OR (NS): 0.93 (0.53 to 1.64); p=0.93	ESRD
		Lys751Gln	Gln: 37.0/37.0				OR (NS): 0.98 (0.55 to 1.74); p=0.94	
	*XRCC1* (NS)	Arg399Gln	Gln: 34.0/19.0				OR (NS): 2.48 (1.36 to 4.52); p=0.002	
Rezk et al. [23]	*Renalase* (10q23.21)	rs2296545	C: 28.7/16.3 C: 29.4/16.3 (hypertensive CKD/controls)	PCR-RFLP	Satisfied HWE (p-value NE)	NS	OR: 2.14 (1.07 to 4.26); p=0.04 OR: 2.10 (1.07 to 4.26); p=0.041 (hypertensive CKD/controls)	CKD Hypertensive CKD
Abdallah et al. [26]	*Renalase* (10q23.21)	rs2576178	G: 56/16	PCR-sequencing; gelelectrophoresis	NS	NS	OR: 7.188 (3.5 to 14.7); p<0.05	ESRD
		rs10887800	G: 26/12				OR: 12.3 (5.6 to 27.1); p<0.05	
Elshamaa et al. [25]	*ADIPOQ* (3q27.3)	rs1501299G>T;	T: 18.6/10.7 T (CT/MHD): 15.6/20.7	PCR-sequencing; gelelectrophoresis	Satisfied HWE (p-value NE)	NS	p=0.04 (TT genotype distribution between cases and controls)	Advanced CKD (ESRD)
		rs2241766T>G	G: 0.0/0.0 G (CT/MHD): 0.0/0.0					
Elhelbawy et al. [24]	*CCR2* (3q21.31)	G190A	G: 75.7/90.0 A: 24.3/10.0	PCR-RFLP	NS	NS	OR: 2.8 (1.40 to 5.51); p<0.05 OR: 4.1 (1.27 to 13.03); p<0.05 OR: 2.9 (1.14 to 7.3); p<0.05	CKD

*ACR* albumin/creatinine ratio, *BMI* body mass index, *CKD* chronic kidney disease, *CKD-EPI* Chronic Kidney Disease Epidemiology Collaboration, *CRF* chronic renal failure, *CT* conservative treatment, *DBP* diastolic blood pressure, *EE* effect estimate, *eGFR* estimated glomerular filtration rate, *ESRD* end-stage renal disease, *HIV* human immunodeficiency virus, *HR* hazard ratio, *HWE* Hardy–Weinberg equilibrium, *MAF* minor allele frequency, *MDRD* Modification of Diet in Renal Disease, *MHD* maintenance hemodialysis, *NO* nitric oxide, *NS* not specified, *OR* odds ratio, *SBP* systolic blood pressure, *UA* unadjusted

### Association of genetic markers with CKD and related traits

According to the studies included in this review, some SNP’s investigated in the *MYH9* [28], *AT1R* [19], and *MTHFR* [20] genes failed to predict prevalent CKD, ESRD or related traits (serum creatinine, eGFR and ACR), while variants in the *APOL1* [27, 29, 30], *apoE* [21], *eNOS* [18, 20], *XPD* [22], *XRCC1* [22], *renalase* [23, 26], *ADIPOQ* [31] and *CCR2* [24] genes were associated with either prevalent CKD or progression of CKD, ESRD, or other surrogate measures of renal function.

The majority of CKD-associated polymorphisms were conducted in single studies. In a Moroccan population, the e4 allele and the E3E4 genotype of the *apoE* gene demonstrated a significant association with ESRD (OR = 0.491; *p* = 0.009 and OR = 0.316, *p* < 0.001, e4 and E3E4, respectively) [21]. However, this association was unadjusted for any potential confounder effects. Both Kerkeni et al. [20] and Elshamaa et al. [18] conducted genetic association studies on SNPs in the *eNOS* gene, adjusting for potential confounders, albeit different population groups and different SNPs. According to Kerkeni et al. [20], the *eNOS* SNP found in exon 7 (G894 T) was an independent risk factor of severity of CKD (*p* = 0.01) in Tunisian adults. Similarly, Elshamaa et al. [18] found the a-allele in the *eNOS* (intron 4) gene to predict ESRD in Egyptian children (*p* < 0.05) [18]. Radwan et al. [22] investigated three polymorphisms in the DNA repair genes (*XPD* and *XRCC1*) and found that patients with *XRCC1–399 Arg/Gln* genotype had a significantly higher risk of developing ESRD (OR: 2.48; 95% CI: 1.36–4.52). Furthermore, the haplotypes containing *XRCC1–399 Arg/Gln* and *XPD-312 Asp/Asn* as well as *XRCC1–399 Arg/Gln* and *XPD-751 Lys/Gln* were significantly associated with the development of ESRD (OR: 8.35, 95%CI: 1.94–35.85, *p* = 0.004 and OR: 9.22, 95%CI: 2.14–39.71, *p* = 0.003, respectively). Two studies, both in Egyptian populations, investigated polymorphisms of the renalase gene [23, 26]. Rezk et al. [23] found that patients with the CC genotype and carriers of C allele of the rs2296545 renalase gene were significantly more likely to have prevalent CKD (CC genotype; OR: 4.84, 95%CI: 1.28–18.2, *p* = 0.02 and C-carrier; OR: 2.14, 95%CI: 1.07–4.26, *p* = 0.04). Abdallah et al. [26], conversely found that carriers of the G allele of the rs2576178 and rs10887800 *renalase* gene were associated with increased risk of developing ESRD (OR: 7.188, 95%CI: 3.5–14.7, *p* < 0.05 and OR: 12.3, 95%CI: 5.6–27.1; *p <* 0.05). However, in both studies no adjustments were made for potential confounders. ADIPOQ+276G > T was also investigated for association with ESRD in Egyptian children [31]. This study suggested that the +276G > T allele may indirectly contribute to CKD susceptibility by increasing adiponectin levels (p = 0.04). Elhelbawy et al. [24] found a significant association between *CCR2*–641 and chronic renal failure, particularly the AG genotype (OR = 2.8, 95% CI = 1.40–5.51), combined AG and GG genotypes (OR = 4.1, 95% CI = 1.27–13.03) and A allele (OR = 2.9, 95% CI = 1.14–7.3).

As seen in Table 2, only polymorphisms in the *APOL1* gene were investigated in more than one ethnic group, with the observed association similar in at least two population groups. Indeed, according to the study conducted in the Yoruba tribe of Nigeria [29], two single *APOL1* SNPs (rs73885319 and rs60910145) were significantly associated with CKD under all genetic models, with the largest effect under the recessive model (OR: 3.85 and 3.12 for rs73885319, and rs60910145, respectively). Furthermore, due to the linkage disequilibrium (D-prime = 1.00, *r*^2^ = 0.82) between the two SNPs, adjusting for either SNP resulted in no association for the other SNP. Similarly, albeit a different population (mixed-race South Africans), Matsha et al. [27] found the same two single SNPs (rs73885319 and rs60910145) to be associated with prevalent CKD, however only under the recessive model (*p* = 0.047) (as measured by the CKD-EPI eGFR equation), even after adjusting for multiple confounders. The study did not observe an association between these single *APOL1* SNPs and any of the other surrogate measures of kidney function. Tayo et al. [29] also investigated the adjusted association of *APOL1* haplotypes, namely the G-A-G haplotype (rs9622363–rs73885319–rs60910145) and the G1 haplotype (rs73885319 and rs60910145) and found both to be significantly associated with CKD under all models of genetic association (G-A-G, ORs: 2.26; *p* = 0.005, OR: 2.54; *p* = 0.023 and OR: 3.79; *p* = 0.041 for the additive, dominant and recessive modes; G1, OR: 2.25; *p* = 0.006, OR: 2.52; *p* = 0.025 and OR: 3.80; p = 0.041 for the additive, dominant and recessive modes). Ulasi et al. [30] also conducted a study on the *APOL1* G1 haplotype (rs73885319 and rs60910145) and G2 (rs71785313) (Wt:G1 or Wt:G2; G1:G1 or G1:G2 or G2:G2) in the Igbo tribe of Nigeria. This study found no significant effect of the Wt:G1 or Wt:G2 one-copy, but observed a high association between *APOL1* two-risk alleles (G1:G1 or G1:G2 or G2:G2) and CKD (OR: 4.8; *p* = 5.1E-03), even after adjusting for various confounders.

## Discussion

To the best of our knowledge, this is the first comprehensive report of the current evidence on genetic polymorphisms associated with renal disease amongst populations in Africa. This review highlights the lack of genetic association studies conducted within the borders of Africa, despite the known genetic link to CKD and the genetic diversity in Africa.

All the studies included in this review used the candidate gene approach, and amongst these, only *MYH9* polymorphisms has been previously investigated by GWAS and showed directional association with CKD in populations elsewhere [10]. Indeed, multiple *MYH9* SNPs have been identified as powerful predictors of non-diabetic kidney disease in African Americans [32], Hispanic-Americans [33], and individuals of European ancestry [34]. However, from this review we found no evidence for the associative role of *MYH9* polymorphisms in non-diabetic CKD patients in Africa, as all eight SNPs investigated in populations from Nigeria and South Africa failed to predict prevalent CKD or any other surrogate measure of kidney function [28, 29]. Differences in linkage disequilibrium structure might however explain the lack of genetic association in studies conducted in these African populations. Indeed, previous studies have shown that the G1 and G2 risk variants of the *APOL1* gene are in strong linkage disequilibrium with variants in *MYH9.* Indeed, most of the association previously attributed to *MYH9* variants or haplotypes with CKD could be explained by their genetic linkage with *APOL1* polymorphisms in populations of African ancestry residing outside the African continent [35, 36]. In contrast, the studies included in this review instead observed independent association between four SNPs of the *APOL1* gene and with either prevalent CKD, serum creatinine, eGFR or ACR in the included studies [27, 29, 30]. This strong association between *APOL1* polymorphisms and non-diabetic kidney disease found in studies in this review have been replicated in several studies [37–45] since the initial findings reported in African Americans [35, 36]. In addition, as reported in all the above mentioned studies, the risk is mostly conferred by the presence of two copies of the risk alleles, that is, homozygous or compound heterozygous compared to no or one *APOL1* risk variant [35, 36]. It would therefore be of great interest if larger population studies are conducted to ascertain the kidney disease-*APOL1* association across African population groups.

Currently, the role of the polymorphisms in the *apoE* [21], *eNOS* [18, 20], *XPD* [22], *XRCC1* [22], *renalase* [23, 26], *ADIPOQ* [31] and *CCR2* [24] genes in the aetiology of CKD remains controversial and further larger studies should be conducted to confirm these results in population groups within Africa. Certainly, various polymorphisms have been associated, both directly and indirectly, with increased CKD risk in certain populations and decreased CKD risk in others or alternatively have no convincing association. This is true for the polymorphisms investigated in the current review. For example, Lahrach et al. [21] showed that the e4 allele and the E3E4 genotype of the *apoE* gene demonstrated a strong association with ESRD, similar to a study conducted in a Swedish population [46]. However, a study conducted in African Americans and European Americans showed an opposite effect, with the e4 allele being associated with decreased risk of ESRD progression and decreased risk of prevalent ESRD [47], with no association found between the e4 allele and CKD in Asian populations [48]. The genetic link between *eNOS* (4a; intron4) and ESRD [18] and CKD severity (G894 T; exon7) [20] have also been studied in two African populations, and in both studies, as in various other studies [49–51], the polymorphisms under investigation were found to be significantly associated with kidney disease. However, this association between polymorphisms of *eNOS* and kidney disease is not fully elucidated, as the direction and magnitude have been found to differ by population and even within the same population. For example, Bellini et al. [52] demonstrated a strong association between *eNOS* 4a polymorphism and ESRD risk in a Brazilian population, while Marson et al. [53] found no significant correlation between *eNOS* 4a polymorphism and ESRD risk in a similar Brazilian population group. The association between DNA repair genes (XPD and XRCC1) and kidney disease is not commonly investigated, and with the exception of the study reviewed in this publication [22], has only been investigated previously in a Turkish population [54]. Both studies showed an association between DNA repair gene polymorphisms and ESRD development. However, the effect estimates amongst the African population were higher than that reported in the Turkish population. From the included studies, it is evident that investigating regional differences in the relationship between genes and CKD risk within Africa has relevance, considering the genetic diversity among ethnic population groups in the continent [55].

Our study has some limitations, which include the small number of existing studies, which precluded statistical analysis by means of meta-analysis. Furthermore, as a result of existing genetic association studies not always reporting on key methodological information that includes testing the HWE, the sample size/power calculations, clear description of controls, consideration and correction for population stratification, as well as the levels of adjustment, it is difficult to draw definitive inferences from these studies. In addition, the sample size of the included studies was much smaller than other studies conducted outside of Africa, thus as a result it is possible that with larger sample sizes, additional previously proposed candidate genes may have reached statistical significance. Indeed, with the largest included study comprising 859 participants [27], it is highly likely that most existing studies on the genetics of kidney disease in Africa have been underpowered to replicate existing loci or estimate effects with precision. Furthermore, the majority of included studies were conducted in Egyptian populations, thus not covering all the scope of genetic variations that exist on the African continent. The age range, which varied from approximately 9–60 years, and the range of covariates included in adjustment of the estimates of association also differed substantially across studies and could possibly affect between-studies comparisons. In addition, since we had no access to individual participant data, refined analyses and accounting for potential confounders and other types of bias, could not be executed. However, despite the shortcomings of this review, the strength resides in the fact that, according to our knowledge, this is the first study to systematically and comprehensively review the existing data on genetic association studies of CKD in the context of Africa.

## Conclusion

The putative genetic risk factors that have emerged from current data represent the most promising kidney disease susceptibility genes described to date in populations within Africa. However, larger-scale genetic association studies are needed to further expand our knowledge of the underlying genetic mechanisms of kidney disease among populations within Africa.

## Additional files

Additional file 1: **Table S1.** Medline (Pubmed) search strategy (from inception to August 2017). (DOCX 20 kb)

Additional file 2: **Table S2.** SCOPUS search strategy (from inception to August 2017). (DOCX 20 kb)

Additional file 3: **Table S3.** EBSCOhost search strategy (from inception to August 2017). (DOCX 20 kb)

Additional file 4: **Table S4.** Web of Science search strategy (from inception to August 2017). (DOCX 20 kb)

## Abbreviations

ACR: Albumin to creatinine ratio
*ADIPOQ*: Gene encoding adiponectin
*apoE*: Gene encoding apolipoprotein E
*APOL1*: Apolipoprotein L1
*AT1R*: Gene encoding angiotensin II receptor type 1
BMI: Body mass index
*CCR2*: Gene encoding C-C chemokine receptor type 2
CKD: Chronic kidney disease
CKD-EPI: Chronic Kidney Disease Epidemiology Collaboration
CRF: Chronic renal failure
CT: Conservative treatment
DBP: Diastolic blood pressure
DM: Diabetes mellitus
EE: Effect estimate
eGFR: Estimated glomerular filtration rate
*eNOS*: Gene encoding endothelial nitric oxide synthase
ESRD: End-stage renal disease
GWAS: Genome-wide association studies
HIV: Human immunodeficiency virus
HR: Hazard ratio
HT: Hypertensive
HWE: Hardy-Weinberg equilibrium
K/DOQI: NKF Kidney Disease Outcomes Quality Initiative
MAF: Minor allele frequency
MDRD: Modification of Diet in Renal Disease
MeSH: Medical Subject Headings
MHD: Maintenance hemodialysis
MRF: Moderate renal failure
*MTHFR*: Gene encoding Methylene tetrahydrofolate reductase
*MYH9*: Gene encoding myosin, heavy chain 9
NO: Nitric oxide
NS: Not specified
NT: Normotensive
OR: Odds ratio
PCR-RFLP: Polymerase chain reaction restriction fragment length polymorphism
SAMRC: South African Medical Research Council
SBP: Systolic blood pressure
SNP: Single nucleotide polymorphisms
SRF: Severe renal failure
SSA: Sub-Saharan Africa
UA: Unadjusted
*XPD*: *G*ene encoding xeroderma pigmentosum group D
*XRCC1*: Gene encoding X-ray repair cross-complementing protein 1
