# Supplementary material for: An African perspective on the genetic risk of chronic kidney disease: a systematic review
Source: BMC Med Genet. 2018 Oct 19;19:187. doi: 10.1186/s12881-018-0702-x (PMC6194564; doi:10.1186/s12881-018-0702-x)
Supplement: Supplementary file 1 — Table S1. Medline (Pubmed) search strategy (from inception to August 2017). (DOCX 20 kb) [file 12881_2018_702_MOESM1_ESM.docx]

**Table S1. Medline (Pubmed) search strategy (from inception to August 2017)**

| **Search** | **Query** | **Number of hits** |
| --- | --- | --- |
| #1 | "chronic kidney disease" OR "chronic kidney failure" OR "chronic renal disease" OR "chronic renal failure" OR "end-stage renal disease" OR "end-stage renal failure" | 130 533 |
| #2 | “serum creatinine” OR eGFR OR “estimated glomerular filtration rate” OR “urinary albumin” | 82 758 |
| #3 | genetics OR "genetic marker" OR "genetic polymorphism" OR "single nucleotide polymorphism" OR polymorphism OR gene OR allele | 3 674 080 |
| #4 | Africa OR African OR "Central Africa" OR "Central African" OR "West Africa" OR "West African" OR "Western Africa" OR "Western African" OR "East Africa" OR "East African" OR "Eastern Africa" OR "Eastern African" | 400 555 |
| #5 | "North Africa" OR "North African" OR "Northern Africa" OR "Northern African" OR "South African" OR "Southern Africa" OR "Southern African" OR "sub Saharan Africa" OR "sub Saharan African" OR "subSaharan Africa" OR "subSaharan African" | 42 293 |
| #6 | Algeria OR Angola OR Benin OR Botswana OR "Burkina Faso" OR Burundi OR "Cabo Verde" OR Cameroon OR "Central African Republic" OR Chad OR Comoros OR "Democratic Republic of the Congo" OR "Republic of the Congo" OR "Cote d'Ivoire" OR “Ivory Coast” | 36 375 |
| #7 | Djibouti OR Egypt OR "Equatorial Guinea" OR Eritrea OR Ethiopia OR Gabon OR “Gabonese Republic” OR Gambia OR Ghana OR “Gold Coast” OR Guinea OR "Guinea-Bissau" | 249 403 |
| #8 | Kenya OR Lesotho OR Basutoland OR Liberia OR Libya OR Jamahiriya OR Jamahiryia OR Madagascar OR “Malagasy Republic” OR Malawi OR Nyasaland OR Mali OR Mauritania OR Mauritius OR Morocco OR Mozambique | 56 186 |
| #9 | Namibia OR Niger OR Nigeria OR Rwanda OR "Sao Tome and Principe" OR “Sao Tome” OR Senegal OR Seychelles OR "Sierra Leone" OR Somalia OR “French Somaliland” OR "South Africa" | 149 061 |
| #10 | "South Sudan" OR Sudan OR Swaziland OR Tanzania OR Togo OR “Togolese Republic” OR Tunisia OR Uganda OR Zaire OR Zambia OR Zimbabwe OR Rhodesia | 71 653 |
| #11 | #1 OR #2 | 200 669 |
| #12 | #4 OR #5 OR #6 OR #7 OR #8 OR #9 OR #10 | 682 177 |
| #13 | #11 AND #3 AND #12 | 754 |
